# Supplementary material for: Insights into the computer-aided drug design and discovery based on anthraquinone scaffold for cancer treatment: A protocol for systematic review
Source: PLoS One. 2023 Sep 1;18(9):e0290948. doi: 10.1371/journal.pone.0290948 (PMC10473489; doi:10.1371/journal.pone.0290948)
Supplement: Attachment — Submitted filename: 20230719_Review_Hui Ming Chua.pdf [file pone.0290948.s002.pdf]

Title: **Insights into the computer aided drug design and discovery based on naturally occurring 9,10-anthraquinone scaffold for cancer treatment: A protocol for systematic review**

Date received: **July 19, 2023**

General comments

The research question outlined in the proposed study is valid and would greatly advance the scientific application of computer-aided drug design & discovery (CADD) as a valuable, efficient, and cost-effective tool in the discovery of new therapeutics for management of various human diseases, and in particular, neoplastic conditions.

The 9,10-anthraquinone moiety is a well-known and established scaffold in medicinal chemistry, and boasts of several naturally occurring, semisynthetic and synthetic compounds, both in the clinic and in development for human medical applications. Some of these have been elaborately cited in the manuscript.

Queries to be addressed by the authors:

Though the manuscript is replete with scientific merit, there are a few queries that need to be clarified by the authors. These are highlighted herein after.

Major query

Reading the title of the protocol together with the body text, it is not clear whether the focus of the study will be specifically on the 9,10-anthraquinone, or generally on the anthraquinones and anthraquinone-like scaffolds. This issue should come out clearly.

For example, under the Search Strategy (Lines 207-221), some of the search terms will undoubtedly retrieve anthraquinone-like scaffolds, some of which will be chemically at variance from the intended 9,10-anthraquinone moiety. Will the proposed PRISMA-P flowchart exclude studies not reporting specifically on 9,10-anthraquinone scaffold?

Minor queries

1. It would be prudent to depict the chemical structures of some of the naturally occurring compounds that contain the 9,10-anthraquinone scaffold such as those mentioned on Line 117.
2. The authors should correct all typographical and grammatical errors in the manuscript.

   Examples:

   a. Line 25: Instead of writing “on the go”, replace with more appropriate phrase such as “an imperative”, or “is essential”, or “is of essence”.
   b. Line 36: Only original studies employed…. Insert the word “that” in between “studies” and “employed” to read: “Only original studies that employed …..
   c. Line 50: Delete the word “as” after “remains”.
   d. Line 83: Delete the words “Among the” that precede popular, and therefore start the sentence with the word “Popular”.
   e. Line 86: Delete the phrase “Apart from the”. Hence start the sentence with “De novo drug design….”
   f. Line 96: Replace the word “synthesizable” with the more accurate term “accessible”.
   g. Line 101: Replace the phrase “such unexplored region” with a more appropriate unambiguous terminology, such as “novel chemotypes”.
   h. Line 148: Since this is a proposal for future planned work, the authors should use future tense. In this case, the verb “aimed” should be replaced with the verb “aims” to reflect that the work is still not yet done but is continuing or planned. Similar instances should be actively checked and corrected throughout the manuscript. For example, Line 208.
   i. Line 159: Ensure that the correct terminology for PRISMA is consistently and accurately written. In this case, correct the word “Report” to read “Reporting”.
   j. Line 226: Should be “titles”, not “tittles”.
   k. Line 247: … and their description…
   l. Line 250: The word “timely” appears ambiguous. Consider replacing with a more appropriate word to suggest “relevant” data, e.g., essential, important, relevant, vital, etc.
   m. Line 273: Should not be “threatened” but “threat”.

n. Line 287 should rather read: … "antitumour activities of anthraquinones are mediated via…"
o. Lines 291-292: Consider rephrasing the highlighted sections as follows; "… been commonly used by researchers as starting points in the design of anticancer drugs in the past few …"
p. Line 303: Rectify to read, "… use of computational software…"
