# Supplementary material for: Insights into the computer-aided drug design and discovery based on anthraquinone scaffold for cancer treatment: A protocol for systematic review
Source: PLoS One. 2023 Sep 1;18(9):e0290948. doi: 10.1371/journal.pone.0290948 (PMC10473489; doi:10.1371/journal.pone.0290948)
Supplement: Attachment — Submitted filename: rebuttal letter_response to reviewer 180823.pdf [file pone.0290948.s003.pdf]

Hui Ming Chua
PAPRSB Institute of Health Sciences,
Universiti Brunei Darussalam
Jalan Tungku Link, BE1410
Brunei Darussalam

Academic Editor
PLOS ONE
Public Library of Science
1160 Battery Street San Francisco,
CA 94111

18 August 2023

Dear Editor,

<u>Re: Insights into the computer aided drug design and discovery based on naturally occurring 9,10-anthraquinone scaffold for cancer treatment: A protocol for systematic review (Manuscript Number: PONE-D-23-19138)</u>

Thank you for providing us with the opportunity to revise our manuscript and for the constructive comments which we believe have greatly enhanced the quality and clarity of our paper. I would like to address the major and minor queries as raised.

Major query:
Focus of the study: We appreciate the reviewer's observation regarding the potential ambiguity between the 9,10-anthraquinone scaffold and other anthraquinone-like scaffolds. To clarify, the primary focus of our study is on anthraquinone, in this case it may include 9,10-anthraquinone, anthraquinone in general or even anthraquinone-like scaffold. In addition, all studies that utilized CADD tools in designing or discovering anticancer drugs containing anthraquinone scaffold, either naturally derived, semi-synthetic or synthetic will be also included in the review. Therefore, the search strategy (Lines 207-221) was developed to retrieve all possible studies in a comprehensive manner by including several different keywords/synonyms for anthraquinone.
In such case, we have revised the title to: "Insights into computer-aided drug design and discovery based on anthraquinone scaffold for cancer treatment: A protocol for systematic review."

Minor queries:
Chemical structures: In light of your suggestion, we have included the chemical structures of the naturally occurring anthraquinone-derivatives as mentioned on Line 117, in which a figure file has been prepared (Fig.1 Naturally Occurring Anthraquinone-derivatives and Their Chemical Structures Depiction).

Typographical and Grammatical Errors: We apologize for the oversight and have thoroughly reviewed the manuscript for typographical and grammatical errors. All instances as pointed out, from Line 25 to Line 303, have been corrected in line with the recommendations.

To provide a few specific examples (by referring to “Revised manuscript with tracked changes):
Line 25: We have replaced “on the go” with “is essential”.
Line 84: The sentence now begins with "Popular".
Line 86: Word 'that' has been added after “approach” should be inserted. Similarly, line 96, after the word compounds. Line 105, replace the word 'considerably' with 'considerable'.
Line 97: Word "synthesizable" has been replaced with "accessible", word “that” has been added after “compounds”.
Line 102: "such unexplored region" has been replaced with "novel chemotypes".
Line 152: The verb "aimed" is replaced with "aims".

We have also ensured consistency in terminology for PRISMA as suggested.

We hope that these revisions address the concerns raised, and that our manuscript now meets the standards for publication in PLOS ONE. Once again, I appreciate the thoughtful feedback, and am available for any further clarifications that might be necessary.

Thank you.

Sincerely,
Hui Ming Chua (corresponding author)
Universiti Brunei Darussalam
